# Mitogenomic Phylogeny and Adaptive Evolution of Snailfishes (Liparidae) Reveal Correlation Between tRNA Rearrangements and Deep-Sea Colonization

**DOI:** 10.3390/biology15040295

**Published:** 2026-02-07

**Authors:** Ruxiang Wang, Ang Li, Shuai Che, Huan Wang, Shufang Liu

**Affiliations:** 1 State Key Laboratory of Mariculture Biobreeding and Sustainable Goods, Yellow Sea Fisheries Research Institute, Chinese Academy of Fishery Sciences, Qingdao 266071, China; 2 College of Environmental Science and Engineering, Ocean University of China, Qingdao 266100, China

**Keywords:** *Liparis chefuensis*, *Liparis tanakae*, mitochondrial genome, Liparidae, phylogeny, gene rearrangement, deepsea adaptation

## Abstract

The snailfish family (Liparidae) represents one of the most rapidly speciating and ecologically diverse lineages of marine fishes, with species distributed across a broad bathymetric range from intertidal zones to the hadal depths. Despite their ecological and evolutionary significance, phylogenetic relationships and adaptive mechanisms within Liparidae remain poorly resolved due to morphological conservatism, phenotypic plasticity, and limited genomic resources due to challenges such as sampling difficulties and a reliance on partial mtDNA markers. In this study, we sequenced, assembled, and annotated the complete mitochondrial genomes of two snailfish species, *Liparis chefuensis* and *Liparis tanakae*, collected from the Yellow Sea. The mitogenome of *L. chefuensis* is 18,870 bp in length, encoding 13 protein-coding genes (PCGs), 2 rRNAs, and 22 tRNAs, while that of *L. tanakae* spans 17,485 bp and contains 13 PCGs, 2 rRNAs, and 23 tRNAs. Phylogenetic reconstruction based on the concatenated sequences of 13 mitochondrial PCGs from 15 liparid species revealed that *L. chefuensis* clusters within the subgenus *Lyoliparis*, contradicting its previous classification under *Careliparis* and suggesting a need for taxonomic reassessment. Notably, we identified distinct patterns of tRNA gene rearrangement in the cluster between *ND2* and *COI*, which suggest a link to both phylogeny and habitat depth. Shallow-water species (<30 m) possess the tRNA^Trp^-tRNA^Tyr^-tRNA^Ala^-tRNA^Asn^-tRNA^Cys^ (WYANC) arrangement, whereas deep-water species (>100 m) display the derived tRNA^Trp^-tRNA^Asn^-tRNA^Cys^-tRNA^Tyr^-tRNA^Ala^-tRNA^Cys^/tRNA^Ala^ (WNCYAC/A) configurations. These rearrangements are hypothesized to originate from tandem duplication events followed by random gene loss, potentially reflecting adaptive evolution to deep-sea environments. Additionally, *L. tanakae* exhibits a markedly higher number of non-canonical G–U and A–C base pairs in its tRNA secondary structures, indicating substantial structural divergence. Our findings not only provide essential mitogenomic resources for snailfish systematics and species identification but also propose that tRNA rearrangements in mitochondrial genomes may serve as genomic innovations facilitating deep-sea colonization. This study enhances our understanding of mitochondrial genome evolution and environmental adaptation in marine fishes.

## Simple Summary

We sequenced and analyzed the complete mitochondrial genomes of two snailfish species, *Liparis chefuensis* and *Liparis tanakae*, from the Yellow Sea. Our study reveals that these fishes show unique rearrangements in their mitochondrial tRNA genes, which are closely linked to their habitat depths. Shallow-water species exhibit one gene order, while deep-water species show a different arrangement. This suggests that changes in mitochondrial gene organization may help these fishes adapt to extreme deep-sea environments. Our findings provide valuable genetic data for snailfish identification and deepen our understanding of how marine organisms evolve to survive in the deep ocean.

## 1. Introduction

The typical vertebrate mitochondrial DNA (mtDNA) is a double-stranded, circular molecule, 15–20 kb in length, usually containing 13 protein-coding genes (PCGs), 2 rRNA genes, 22 tRNA genes, and 2 main non-coding regions: the control region (D-loop) and the origin of light-strand replication (O_L_) [1]. Fish mitochondrial genomes are generally highly conserved in structure, particularly in gene order, which is a key reason for the widespread use of mtDNA as a molecular marker in fish systematics (e.g., for population identification, phylogeography, evolution, and phylogenetic reconstruction) [2,3,4,5]. While advancements in sequencing technology and the expansion of genomic databases have led to increased reporting of mitochondrial gene rearrangements in fishes [6,7], these events remain relatively rare across the group. Although such rearrangements have now been documented in at least 34 teleost families, they collectively represent a small proportion of known species diversity and are often concentrated within specific evolutionary lineages, such as the order Anguilliformes, Notothenioidei and the genus *Parupeneus* [7,8,9,10]. Mitochondrial gene rearrangements can harbor significant phylogenetic information, as demonstrated in numerous terrestrial vertebrates [11,12,13,14]. In contrast, reported cases in teleost fish remain limited and taxonomically restricted; consequently, studies utilizing gene rearrangements to investigate fish systematics and evolution are still relatively rare.

Mitochondria are central to cellular energy metabolism, producing ATP via the electron transport chain—a process dependent on all 13 protein-coding genes encoded in the mitochondrial DNA (mtDNA) [15]. Functional constraints on these mtDNA genes have been shown to influence adaptation across diverse environments, including those related to locomotion, climate, and elevation [16,17,18]. Of particular relevance to deep-sea colonization, the extreme conditions of high hydrostatic pressure, low temperature, and often limited oxygen availability impose strong selective pressures on energy metabolism [19]. Consequently, adaptive evolution in mitochondrial function has been considered as a key mechanism enabling survival in the deep sea, highlighting the critical link between mtDNA evolution and adaptation to abyssal environments [15].

The snailfish family (Liparidae; Teleostei: Scorpaeniformes: Cottoidei) exhibits extremely high species diversity, comprising 32 genera and over 520 species, making it one of the most rapidly speciating lineages of marine fishes [20,21,22]. Snailfishes are globally distributed, inhabiting depths from the intertidal to the hadal zone in temperate and cold regions, primarily concentrated in the North Pacific, North Atlantic, and polar seas. They are also found in deep-sea bottoms of tropical and subtropical regions near the equator [23,24,25,26]. The two most diverse genera, *Careproctus* and *Paraliparis*, are distributed in both Hemispheres, while the genus *Liparis* is restricted to the Northern Hemisphere [26]. North Pacific waters, particularly near Alaska, harbor an extraordinary diversity of Liparidae, with over 85 species described or known but awaiting formal description [27,28,29]. The discovery and description of new species and new geographic records are ongoing [30,31,32,33,34,35]. However, due to high morphological similarity, phenotypic plasticity, rapid evolutionary rates, broad distributions, and difficulties in obtaining deep-sea samples, taxonomic research on Liparidae has progressed slowly, and the phylogenetic relationships of many species remain controversial [26].

Currently, complete mitochondrial genome data are publicly available for only 13 liparid species [36,37,38], representing merely 2.5% of the family’s diversity, indicating a significant data gap. *Liparis chefuensis* and *Liparis tanakae* both belong to the genus *Liparis*. The former is endemic to the Yellow Sea residing in areas shallower than 30 m [39], while the latter is the only dominant liparid species in Chinese waters residing around 100–121 m [40], making both significant for regional biodiversity studies. However, the lack of complete and accurate mitochondrial genome sequences for these species has impeded phylogenetic and evolutionary studies of Liparidae. Therefore, it is necessary to sequence and analyze their complete mitochondrial genomes to supplement and improve the family’s genomic database.

In summary, this study aims to:

1. Sequence, assemble, and annotate the complete mitochondrial genomes of *L. chefuensis* and *L. tanakae*, analyzing their structural characteristics (base composition, codon usage bias, tRNA structure);
2. Construct phylogenetic trees based on mitochondrial PCGs from 15 Liparidae species to clarify their evolutionary relationships;
3. Investigate tRNA rearrangement in the mitochondrial genomes of the genus *Liparis*, explore their association with phylogeny and habitat depth, and analyze potential formation mechanisms and functional impacts.

Our results provide essential data for the biological research of *L. chefuensis* and *L. tanakae*, re-examine liparid phylogenetic relationships, and offer new insights into the deep-sea adaptation mechanisms within the family.

## 2. Materials and Methods

### 2.1. Sample Collection, DNA Extraction, and Quality Assessment

*Liparis chefuensis* was collected from the intertidal zone of the Yellow River Estuary in July 2022 at a depth of 4 m and *Liparis tanakae* was collected during an autumn survey cruise in the Yellow Sea in September 2021 at a depth of about 110 m. Due to logistical constraints during the research cruise, samples were temporarily stored at −20 °C for the 5–7 day transit period. Upon return, muscle tissue was immediately dissected for DNA extraction.

Genomic DNA was extracted using a TIANamp Marine Animal DNA Kit (Tiangen, Beijing, China). DNA integrity and quality were assessed by 1% agarose gel electrophoresis, and concentration was detected using a Nano-300 nano spectrophotometer (Allsheng, Hangzhou, China).

### 2.2. Library Construction, Sequencing, and Data Filtering

Libraries were constructed and sequenced on the DNBSEQ platform (MGI, Shenzhen, China). Briefly, DNA was sheared by ultrasonication into 300–400 bp fragments, end-repaired, and ligated to adapters. Following PCR amplification and circularization of the products, libraries were sequenced. Library construction and sequencing were performed by the BGI Genomics Co., Ltd. (Shenzhen, China).

Raw sequencing data with about 45X average depth were processed with SOAPnuke (v1.5.3) [41] to remove adapter sequences, reads shorter than 150 bp, reads with polyX length exceeding 50 bp, reads with N content exceeding 1%, and other low-quality reads, yielding clean data for subsequent assembly.

### 2.3. Mitochondrial Genome Assembly and Annotation

De novo assembly of the mitochondrial genome was performed using Novoplasty (v4.3.5) [42], with K-mer set to 33 and using the mitochondrial *COI* gene sequence of *L. chefuensis* and *L. tanakae* as a seed (a common choice given its high conservation and utility for anchoring vertebrate mitogenome assemblies). Annotation and visualization of the mitochondrial genome were conducted on MitoFish (v2025.06) [43] using the vertebrate mitochondrial genetic code.

MEGA X (v10.2) [44] was used for base composition statistics and base bias calculation, where GC-skew = (G − C)/(G + C) and AT-skew = (A − T)/(A + T). EMBOSS (v6.6.0) [45] was used for codon usage bias analysis of the whole genome, tRNAs, rRNAs, the control region, and protein-coding genes.

MITOS2 (v2.1.10) [46] was used for annotation and secondary structure prediction of mitochondrial tRNAs, and visualization was performed using VARNA (v3-93) [47].

### 2.4. Phylogenetic Analysis

Complete mitochondrial genome sequences and annotation files for 13 additional Liparidae species were downloaded from NCBI (Table 1). To ensure consistent and accurate annotation, all downloaded sequences were re-annotated using MitoFish (v2025.06) [43] with the vertebrate mitochondrial genetic code and compared against the original annotations. The 13 PCG sequences for each species were extracted, stop codons were removed, and the genes were concatenated per species to create a dataset for phylogenetic analysis. MAFFT (v7.487) [48] was used for sequence alignment of the concatenated PCGs dataset. IQ-Tree (v2.4.0) [49] was used to construct the ML tree with a codon position partition scheme, automatically selecting the best-fit evolutionary model (GTR + F + I + G4), with parameters -bb 1000 and -alrt 1000 for 1000 ultrafast bootstrap replicates to assess branch support and SH-like aLRT test for branch reliability, respectively. PAUP* (v4.0a169) and MrModeltest2 (v.2.4) were used to find the best model for BI analysis. MrBayes (v3.2.7a) [50] was used to construct the BI tree under the best model GTR + I + G, running 2 independent MCMC chains for 107 generations, sampling every 1000 generations, discarding the first 25% as burn-in, ensuring the average standard deviation of split frequencies was <0.01. *Cottus dzungaricus* was selected as the outgroup, that belongs to the suborder Cottoidei, and includes the family Liparidae.

## 3. Results

### 3.1. Mitochondrial Genome Sequencing and Assembly

**Figure 1:**
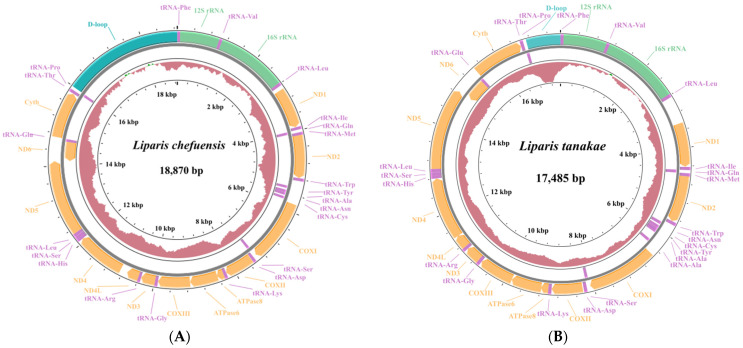
Circular maps of the mitochondrial genomes of *Liparis chefuensis* (**A**) and *Liparis tanakae* (**B**). The outer and inner circles represent genes encoded by the heavy strand and light strand (dark green: D-loop, light green: rRNA, orange: PCGs, pink: tRNA), respectively. The small interior circles indicate GC skew (red: positive, green: negative).

Sequencing of *L. chefuensis* yielded 40,080,591 clean reads, with a Q20 of 97.92%. The final assembled mitochondrial genome was 18,870 bp in length (GenBank accession: PX718959), containing 37 genes: 13 PCGs, 22 tRNA genes, 2 rRNA genes (12S rRNA and 16S rRNA), and 2 main non-coding regions (D-loop and O_L_) (Figure 1A).

Sequencing of *L. tanakae* yielded 48,034,225 clean reads, with a Q20 of 98.18%. The final assembled mitochondrial genome was 17,485 bp in length (GenBank accession: PX718960), containing 38 genes: 13 PCGs, 23 tRNA genes, 2 rRNA genes, and 2 main non-coding regions. Compared to the typical vertebrate mitochondrial genome, it possesses one extra tRNA gene, which is a novel structural feature (Figure 1B).

### 3.2. Phylogenetic Analysis

**Figure 2:**
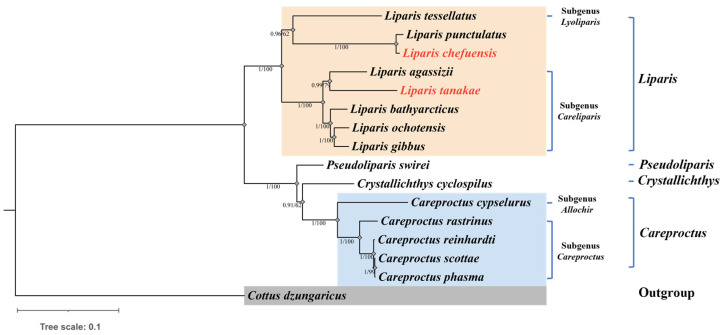
Phylogenetic relationships of Liparidae based on 13 mitochondrial protein-coding genes (red font: *L. chefuensis* and *L. tanakae*). The Maximum Likelihood (ML) and Bayesian Inference (BI) trees are shown. Numbers at branches represent ML bootstrap support values (right) and BI posterior probabilities (left).

The ML and BI trees based on 13 PCGs from 15 Liparidae species showed congruent topologies (Figure 2). Species of the genus *Liparis* formed a monophyletic clade, while species of *Pseudoliparis*, *Crystallichthys*, and *Careproctus* together formed a sister clade. Within the *Liparis* clade, the subgenus *Lyoliparis* (represented by *L. tessellatus*) clustered with *L. punctulatus* and *L. chefuensis*, while species of the subgenus *Careliparis* formed a distinct cluster.

Although some internal nodes received moderate support (Bootstrap < 95%, Posterior Probability < 0.95), the consistent topology between ML and BI analyses suggests the current subgeneric classification of *L. chefuensis* may require re-evaluation.

### 3.3. Mitochondrial Genome Structure Comparison and tRNA Rearrangement

**Figure 3:**
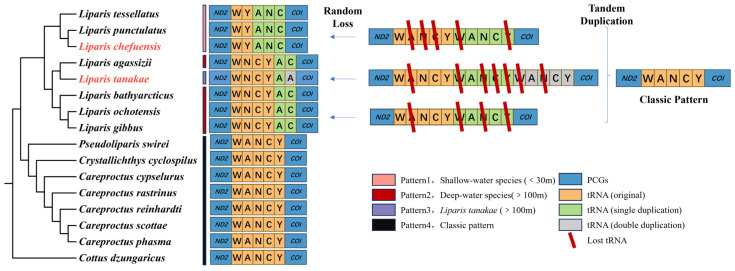
Mitochondrial tRNA gene rearrangements and proposed evolutionary mechanism in Liparidae (red font: *L. chefuensis* and *L. tanakae*). Pink indicates Pattern 1, containing shallow-water species; red indicates Pattern 2, containing deep-water species; purple indicates Pattern 3, containing only the deep-water species *L. tanakae* and the black indicates the Classic pattern. Blue represents *ND2* and *COI* genes. Orange, green, and gray represent tRNA genes (indicated by the single-letter abbreviation of their corresponding amino acid). Red line represents genes randomly lost during evolution.

Comparative analysis revealed tRNA gene rearrangements in the tRNA gene cluster region between *ND2* and *COI* in all eight studied *Liparis* species, displaying three distinct patterns (Figure 3):

1. Pattern 1 (Pink branch): Found in shallow-water species (<30 m: *L. chefuensis*, *L. punctulatus*, *L. tessellatus*), with the order tRNA^Trp^-tRNA^Tyr^-tRNA^Ala^-tRNA^Asn^-tRNA^Cys^ (WYANC);
2. Pattern 2 (Red branch): Found in deep-water *Careliparis* species (>100 m: *L. agassizii*, *L. bathyarcticus*, *L. ochotensis*, *L. gibbus*), with the order tRNA^Trp^-tRNA^Asn^-tRNA^Cys^-tRNA^Tyr^-tRNA^Ala^-tRNA^Cys^ (WNCYAC);
3. Pattern 3 (Purple branch): Unique to the deep-water *Careliparis* species *L. tanakae* (100–121 m), with the order tRNA^Trp^-tRNA^Asn^-tRNA^Cys^-tRNA^Tyr^-tRNA^Ala^-tRNA^Ala^ (WNCYAA);
4. Typical Pattern (Black branch): Genera outside Liparis (*Pseudoliparis*, *Crystallichthys*, *Careproctus*) retained the classic vertebrate tRNA^Trp^-tRNA^Ala^-tRNA^Asn^-tRNA^Cys^-tRNA^Tyr^ (WANCY) arrangement.

Based on the Tandem Duplication and Random Loss (TDRL) model, these patterns can be plausibly explained (Figure 3): The ancestral WANCY cluster likely underwent one or two tandem duplications, followed by random loss of specific genes, giving rise to the observed arrangements. For instance, Pattern 1 could arise from a single duplication followed by loss of the ANC, W, and Y copies. Pattern 2 might result from a single duplication with loss of A, W, N, and Y copies. The complex Pattern 3 in *L. tanakae* may require two duplications followed by loss of A, W, NCYW, and NCY copies.

### 3.4. tRNA Structure Analysis

**Figure 4:**
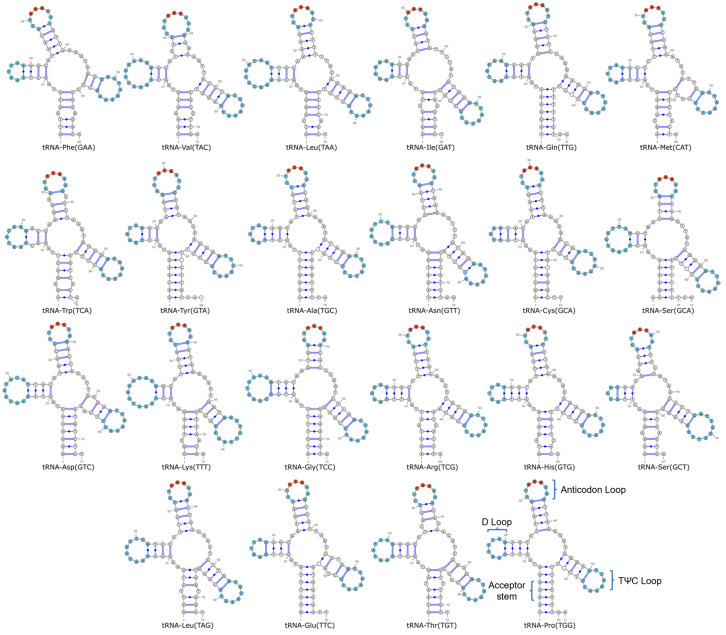
Predicted secondary structures of the 22 tRNAs of *Liparis chefuensis*. The D-loop, TΨC loop, and anticodon loop are highlighted in blue. The anticodon is marked by a red dot.

The 22 tRNAs of *L. chefuensis* had a total length of 1549 bp (66–74 bp per tRNA) and exhibited typical cloverleaf secondary structure (Figure 4). Non-Watson-Crick pairs included 29 G-U and 9 A-C pairs.

**Figure 5:**
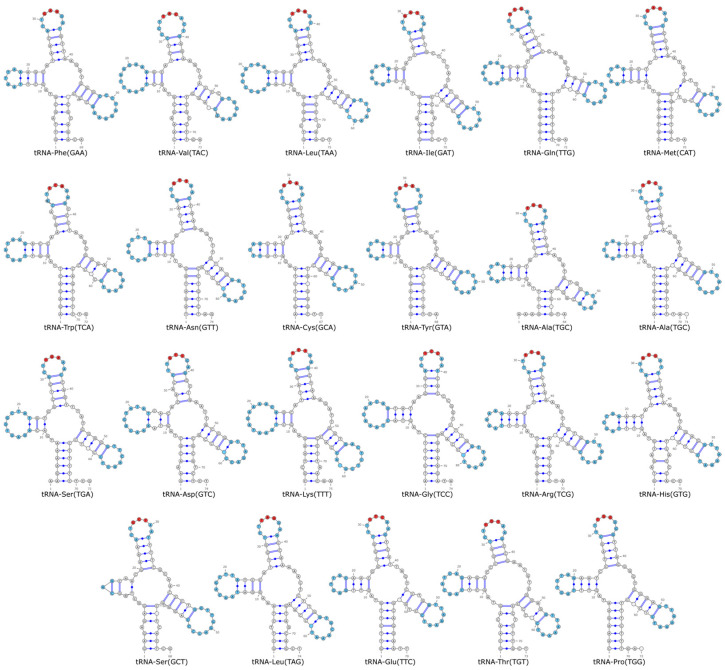
Predicted secondary structures of the 23 tRNAs of *Liparis tanakae*. The D-loop, TΨC loop, and anticodon loop are highlighted in blue. The anticodon is marked by a red dot.

The 23 tRNAs of *L. tanakae* had a total length of 1612 bp (63–74 bp per tRNA) and also formed typical cloverleaf structures (Figure 5). Notably, they contained significantly more non-standard pairs: 44 G-U and 25 A-C pairs.

### 3.5. General Mitochondrial Genome Features

Detailed structural features and nucleotide composition for *L. chefuensis* and *L. tanakae* are provided in Table 2, Table 3, Table 4 and Table 5. Both genomes showed negative GC-skew and positive AT-skew, and an A + T content higher than G + C content, consistent with typical vertebrate mitochondrial genomes.

### 3.6. Codon Usage Bias

Except for *COXI* (start GTG), all PCGs in both species used ATG as the start codon. Stop codon usage varied, with incomplete stop codons (T- or TA-) likely completed to TAA via polyadenylation [51].

**Figure 6:**
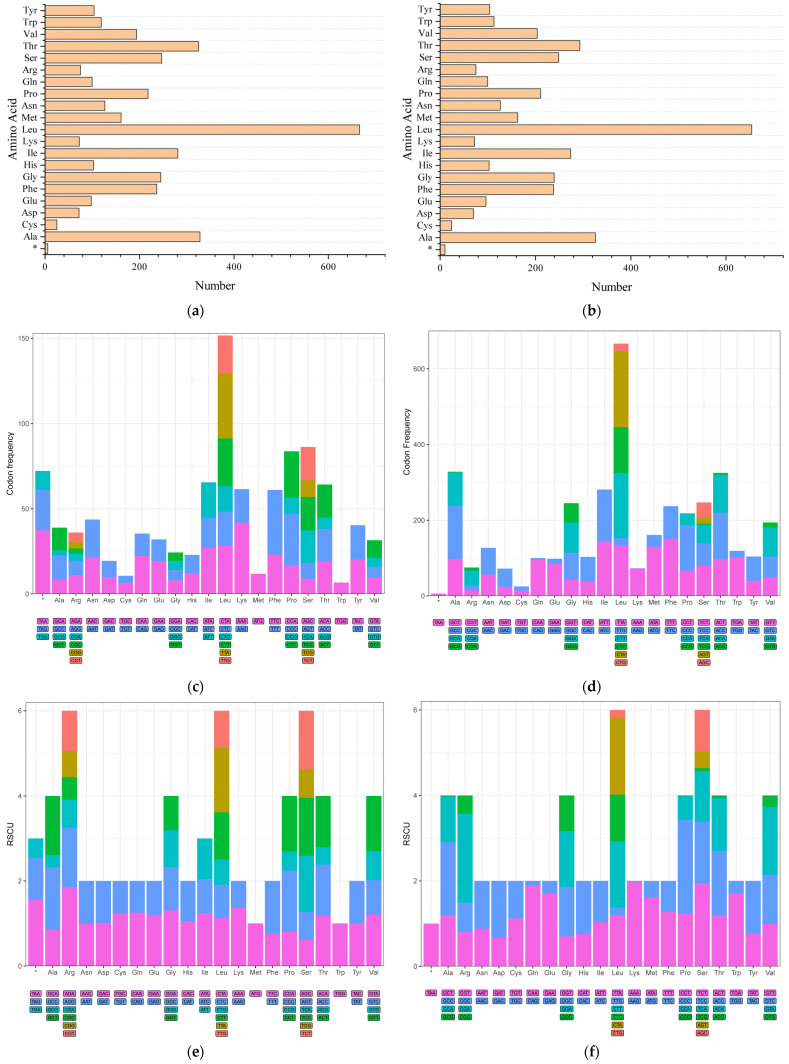
Codon usage analysis for *Liparis chefuensis* and *Liparis tanakae*. (**a**,**d**) Amino acid usage frequency. (**b**,**e**) Synonymous codon usage frequency. (**c**,**f**) Relative Synonymous Codon Usage (RSCU) values for the 13 mitochondrial PCGs. Asterisks (*) denote stop codons.

Relative Synonymous Codon Usage (RSCU) analysis indicated a preference for A/T-ending codons and an avoidance of G/C-ending codons in both species (Figure 6). Leucine, Alanine, and Threonine were the most abundant amino acids.

## 4. Discussion

### 4.1. Phylogenetic Relationships and Taxonomic Implications

Our phylogenetic analysis provides new insights into liparid systematics. The distinct clustering of *Liparis* species separate from *Pseudoliparis*, *Crystallichthys*, and *Careproctus* is consistent with some previous studies using *COI*, RAD-seq, or morphology [18,52,53,54,55]. However, our results place *L. chefuensis* within the *Lyoliparis* clade, contradicting its previous classification in *Careliparis* [26]. This discrepancy may stem from past misidentification, potentially due to the sympatric distribution and morphological similarity between *L. chefuensis* and *L. tanakae* in the Yellow Sea. Morphometric data support this reclassification: the fin ray counts of *L. chefuensis* (dorsal 36–38, anal 29–31, pectoral 34–36 [40]) differ significantly from the characteristics ranges of *Careliparis* (dorsal 39–48, anal 31–37, pectoral 35–46 [55]) and do not fully align with *Lyoliparis* either. Therefore, the subgeneric placement of *L. chefuensis* warrants further investigation using additional molecular markers (e.g., nuclear genes) and detailed morphological re-examination.

### 4.2. tRNA Gene Rearrangements: A Putative Adaptive Innovation

While mitochondrial gene order is generally conserved in fishes, rearrangements are increasingly being documented, often in specific clusters like the one between *ND2* and *COI* [4,10,12,13,56,57,58,59,60,61,62,63]. We identified a novel and phylogenetically correlated rearrangement of the WANCY tRNA cluster in *Liparis*. We hypothesize that the shift from the WYANC pattern observed in shallow-water species to the WNCYAC pattern found in deep-water species represents an important genomic innovation potentially link to adaptation to the deep-sea environment.

#### 4.2.1. Phylogenetic Signal of Rearrangements

Mitochondrial gene rearrangements often contain phylogenetic information. The rearrangement patterns are highly consistent with the phylogenetic relationships within the genus *Liparis*: the subgenus *Lyoliparis* and its closely related species branch exhibit the WYANC arrangement, the subgenus *Careliparis* exhibits the WNCYA + A/C arrangement, while the three genera *Pseudoliparis*, *Crystallichthys*, and *Careproctus* conform to the typical vertebrate WANCY arrangement. Both the rearrangement pattern and the PCGs phylogenetic tree demonstrate the close relationship between *L. chefuensis* and the subgenus *Lyoliparis*, correcting the previous taxonomic placement within the subgenus *Careliparis*.

The perfect congruence between rearrangement patterns and the major phylogenetic clades within *Liparis* underscores the utility of mitochondrial gene order as a phylogenetic marker. The shared derived state (WNCYAC) unites the deep-water *Careliparis* species, while the distinct state (WYANC) characterizes the shallow-water clade. This provides independent evidence for the reclassification of *L. chefuensis*. However, caution is needed, as convergent rearrangements can occur [64], and more data from related taxa are essential.

#### 4.2.2. Correlation with Habitat Depth and Putative Function

The biological function and significance of mitochondrial gene rearrangement phenomena remain unclear but may be related to the action of natural selection in specific habitats [12,52]. The gene rearrangement phenomenon in the genus *Liparis* might provide insights for related research: The genus *Liparis* is generally considered to comprise shallow-water fishes, but different subgenera exhibit significant differences in habitat depth: the subgenus *Liparis* (e.g., *L. montagui* and *L. liparis* [65]) are mostly distributed in shallow waters from 0–100 m [52]; whereas the subgenus *Careliparis* species tend towards deep-sea life, with most distributed in the mesopelagic zone at 400–800 m (e.g., *L. bathyarcticus* primarily inhabits 400–647 m [55], *L. ochotensis* has a depth limit of 761 m [66], *L. gibbus* has a depth limit of 647 m [39]), and a few in the 100–400 m transition zone (e.g., *L. agassizii* and *L. tanakae* have depth limits around 100–121 m [39]). These deep-water *Careliparis* species exhibit the derived WNCYAC rearrangement pattern. Conversely, species inhabiting depths shallower than 30 m (e.g., the subgenus *Lyoliparis* species *L. tessellatus*, *L. punctulatus* [39], and *L. chefuensis* [40]) all possess the WYANC arrangement.

Thus, we can see that, the correlation between rearrangement patterns and habitat depth is striking. Deep-sea conditions (high pressure, low temperature, hypoxia, low energy) impose extreme demands on energy metabolism. Mitochondria, as cellular power plants, are central to meeting these demands. We hypothesize that the derived gene arrangements (WNCYAC, WNCYAA) in deep-water *Liparis* species may confer a putative selective advantage by optimizing mitochondrial function. This optimization could occur through increased transcriptional efficiency, enhanced RNA stability, or altered interactions with nuclear-encoded factors, ultimately boosting energy production under extreme conditions. It is crucial to note that this correlation, while compelling, does not establish causality. The proposed adaptive significance of the tRNA rearrangements remains a hypothesis requiring functional validation.

#### 4.2.3. The Unique Case of *Liparis tanakae*

According to the TDRL model, the formation process of the gene rearrangement in *L. tanakae* is species specific: compared to gene rearrangements in other Liparidae species, *L. tanakae* might have undergone one additional gene cluster duplication, resulting in the unique WNCYAA rearrangement pattern. This might be related to its unique adaptation to the Yellow Sea environment, making it the only dominant species of Liparidae in the Yellow Sea region.

Furthermore, the tRNA secondary structures of *L. chefuensis* contained 29 G-U pairs and 9 A-C non-standard pairs. *L. tanakae* had even more, with 44 G-U pairs and 25 A-C pairs. Although G-U pairs are non-standard, their stability is higher than other non-Watson-Crick pairs and they might represent intermediate states of compensatory mutations, playing an important role in maintaining RNA structure and function [55,56]. The elevated number of non-standard pairs in the tRNAs of *L. tanakae* suggests that its mitochondrial tRNAs have undergone notable structural changes during evolution. Such structural changes could potentially influence tRNA stability and might thereby contribute to adaptive capacity in complex marine environments.

This unique genomic structure, coupled with its exceptionally high number of tRNA non-standard base pairs (which may affect stability and function), might reflect a specialized or transitional adaptive state.

A limitation of this study is the lack of data from the nominal subgenus *Liparis*, typically comprising shallow-water species. Obtaining their mitochondrial genomes is crucial for robustly testing the hypothesis that WYANC is the ancestral shallow-water state.

## 5. Conclusions

We present the first complete mitochondrial genomes for *Liparis chefuensis* and *Liparis tanakae*. Phylogenomic analysis supports the reassignment of *L. chefuensis* to the subgenus *Lyoliparis*. Most significantly, we discovered phylogenetically correlated tRNA gene rearrangements within *Liparis* that are strongly associated with habitat depth. We propose the hypothesis that these rearrangements are not merely neutral markers but may represent genomic adaptations that enhance mitochondrial function, may facilitate the colonization of the deep sea.

This study provides fundamental genetic resources for snailfish research and opens new avenues for investigating the role of mitogenomic architecture in extreme environment adaptation. Future work should focus on: (1) Filling taxonomic gaps, especially sequencing the subgenus *Liparis*; (2) Broadening taxonomic sampling to test the generality of the depth-rearrangement correlation; (3) Elucidating the molecular mechanisms driving these rearrangements; (4) Employing integrated multi-omics approaches (comparative genomics, transcriptomics, proteomics) and physiological assays to directly test the functional consequences of these rearrangements on mitochondrial performance; and (5) future studies could incorporate comparative analyses of non-coding regions (e.g., the D-loop) to elucidate the mechanisms and evolutionary implications of the substantial size variation observed in liparid mitogenomes.

## Figures and Tables

**Table 1:** Sequence information for all species used in phylogenetic analysis.

Genus	Species	Length (bp)	Accession Number
*Liparis*	*Liparis agassizii*	17,896	KX156765.1
	*Liparis bathyarcticus*	17,358	NC_063119.1
	*Liparis chefuensis*	18,870	PX718959
	*Liparis gibbus*	18,466	CM082632.1
	*Liparis ochotensis*	17,522	MG718032.1
	*Liparis punctulatus*	16,771	LC493935.1
	*Liparis tanakae*	17,485	PX718960
	*Liparis tessellatus*	16,447	MK182380.1
*Careproctus*	*Careproctus cypselurus*	16,140	LC493936.1
	*Careproctus phasma*	15,707	OR582698.1
	*Careproctus rastrinus*	15,284	MW401763.1
	*Careproctus reinhardti*	18,218	PV357204.1
	*Careproctus scottae*	15,707	OR582695.1
*Crystallichthys*	*Crystallichthys cyclospilus*	15,711	OR582692.1
*Pseudoliparis*	*Pseudoliparis swirei*	16,593	NC_063120.1
*Cottus*	*Cottus dzungaricus*	16,525	MT897993.1

**Table 2:** Nucleotide composition and base bias in the mitochondrial genome of *L. chefuensis*.

Region	T%	C%	A%	G%	AT%	GC Skew%	AT Skew%
PCGs	33.08	26.61	25.95	14.37	59.03	−0.29868	−0.12079
rRNA	22.77	24.74	33.26	19.23	56.03	−0.12531	0.18722
tRNA	28.41	20.21	29.57	21.82	57.98	0.03831	0.02001
Control region	31.94	13.71	43.25	11.10	75.19	−0.1052	0.15042
Genome	30.16	24.79	30.75	14.30	60.91	−0.26836	0.00969

**Table 3:** Nucleotide composition and base bias in the mitochondrial genome of *L. tanakae*.

Region	T%	C%	A%	G%	AT%	GC Skew%	AT Skew%
PCGs	30.52	26.21	26.21	14.43	56.73	−0.28986	−0.07597
rRNA	22.72	24.32	32.48	20.48	55.20	−0.08571	0.17681
tRNA	28.00	20.27	29.17	22.56	57.17	0.05346	0.02047
Control region	36.29	20.40	42.78	0.53	79.07	−0.94935	0.08208
Genome	27.77	28.10	29.64	14.49	57.41	−0.31956	0.03257

**Table 4:** Genome features of *L. chefuensis*.

Gene	Strand	Location	Size (bp)	Intergenics Length	Anticodon	Amino Acids	Start Codon	Stop Codon
*tRNA^Phe^*	+	1–68	68	0	GAA			
*12S rRNA*	+	69–1011	943	0				
*tRNA^Val^*	+	1012–1083	72	0	TAC			
*16S rRNA*	+	1084–2771	1688	0				
*tRNA^Leu^*	+	2772–2845	74	89	TAA			
*ND1*	+	2935–3909	975	3		324	ATG	TAA
*tRNA^Ile^*	+	3913–3981	69	−1	GAT			
*tRNA^Gln^*	−	3981–4051	71	−1	TTG			
*tRNA^Met^*	+	4051–4119	69	0	CAT			
*ND2*	+	4120–5165	1046	0		348	ATG	TA-
*tRNA^Trp^*	+	5166–5236	71	171	TCA			
*tRNA^Tyr^*	−	5408–5474	67	48	TGC			
*tRNA^Ala^*	−	5523–5591	69	1	GTT			
*tRNA^Asn^*	−	5593–5665	73	36	GCA			
*tRNA^Cys^*	−	5702–5767	66	46	GTA			
*COXI*	+	5814–7358	1545	6		514	GTG	TAA
*tRNA^Ser^*	−	7365–7435	71	3	TGA			
*tRNA^Asp^*	+	7439–7511	73	6	GTC			
*COXII*	+	7518–8208	691	0		230	ATG	T-
*tRNA^Lys^*	+	8209–8282	72	1	TTT			
*ATPase8*	+	8284–8451	168	−8		55	ATG	TAA
*ATPase6*	+	8442–9124	683	0		227	ATG	TA-
*COXIII*	+	9125–9909	785	0		261	ATG	TA-
*tRNA^Gly^*	+	9910–9982	71	0	TCC			
*ND3*	+	9983–10,331	349	0		116	ATG	T-
*tRNA^Arg^*	+	10,332–10,400	69	0	TCG			
*ND4L*	+	10,401–10,697	297	−7		98	ATG	TAA
*ND4*	+	10,691–12,072	1382	0		460	ATG	T-
*tRNA^His^*	+	12,073–12,141	69	0	GTG			
*tRNA^Ser^*	+	12,142–12,208	68	3	GCT			
*tRNA^Leu^*	+	12,212–12,284	73	0	TAG			
*ND5*	+	12,285–14,123	1839	−2		612	ATG	TAG
*ND6*	−	14,120–14,641	522	0		173	ATG	TAG
*tRNA^Glu^*	−	14,642–14,710	69	4	TTC			
*Cytb*	+	14,715–15,851	1137	3		378	ATG	AGA
*tRNA^Thr^*	+	15,855–15,926	72	−1	TGT			
*tRNA^Pro^*	−	15,926–15,995	70	0	TGG			

**Table 5:** Genome features of *L. tanakae*.

Gene	Strand	Location	Size (bp)	Intergenics Length	Anticodon	Amino Acids	Start Codon	Stop Codon
*tRNA^Phe^*	+	1–68	68	0	GAA			
*12S rRNA*	+	69–1012	944	0				
*tRNA^Val^*	+	1013–1084	72	0	TAC			
*16S rRNA*	+	1085–2772	1688	0				
*tRNA^Leu^*	+	2773–2846	74	600	TAA			
*ND1*	+	3447–4421	975	3		324	ATG	TAA
*tRNA^Ile^*	+	4425–4493	69	−1	GAT			
*tRNA^Gln^*	−	4493–4563	71	−1	TTG			
*tRNA^Met^*	+	4563–4631	69	0	CAT			
*ND2*	+	4632–5677	1046	0		348	ATG	TA-
*tRNA^Trp^*	+	5678–5748	71	54	TCA			
*tRNA^Asn^*	−	5803–5875	73	36	GTT			
*tRNA^Cys^*	−	5912–5977	66	1	GCA			
*tRNA^Tyr^*	−	5979–6045	67	−4	GTA			
*tRNA^Ala^*	−	6042–6104	63	198	TGC			
*tRNA^Ala^*	−	6303–6371	69	160	TGC			
*COXI*	+	6532–8076	1545	6		514	GTG	TAA
*tRNA^Ser^*	−	8083–8153	71	3	TGA			
*tRNA^Asp^*	+	8157–8229	73	22	GTC			
*COXII*	+	8252–8942	691	0		230	ATG	T-
*tRNA^Lys^*	+	8943–9016	74	1	TTT			
*ATPase8*	+	9018–9185	168	−10		55	ATG	TAA
*ATPase6*	+	9176–9858	683	0		227	ATG	TA-
*COXIII*	+	9859–10,643	785	0		261	ATG	TA-
*tRNA^Gly^*	+	10,644–10,716	73	0	TCC			
*ND3*	+	10,717–11,065	349	0		116	ATG	T-
*tRNA^Arg^*	+	11,066–11,134	69	0	TCG			
*ND4L*	+	11,135–11,431	297	−7		98	ATG	TAA
*ND4*	+	11,425–12,805	1381	0		460	ATG	T-
*tRNA^His^*	+	12,806–12,874	69	0	GTG			
*tRNA^Ser^*	+	12,875–12,941	67	3	GCT			
*tRNA^Leu^*	+	12,945–13,017	73	0	TAG			
*ND5*	+	13,018–14,856	1839	−4		612	ATG	TAA
*ND6*	−	14,853–15,374	522	0		173	ATG	TAA
*tRNA^Glu^*	−	15,375–15,443	69	4	TTC			
*Cytb*	+	15,448–16,588	1141	0		380	ATG	T-
*tRNA^Thr^*	+	16,589–16,660	72	−1	TGT			
*tRNA^Pro^*	−	16,660–16,729	70	0	TGG			

## Data Availability

The mitochondrial genome supporting this study has been deposited in GenBank (http://www.ncbi.nlm.nih.gov) (accessed on 17 December 2025) under the accession number PX718959-PX718960.

## Abbreviations

The following abbreviations are used in this manuscript:

PCGs	Protein-Coding Genes
O_L_	Origin of Light-strand replication
mtDNA	mitochondrial DNA
ML	Maximum Likelihood
BI	Bayesian Inference
TDRL	Tandem Duplication and Random Loss
RSCU	Relative Synonymous Codon Usage
